# New concepts in ANCA detection and disease classification in small vessel vasculitis: the role of ANCA antigen specificity

**DOI:** 10.31138/mjr.29.1.17

**Published:** 2018-03-19

**Authors:** Elena Csernok

**Affiliations:** Department of Internal Medicine, Rheumatology and Immunology, Vasculitis-Center Tübingen-Kirchheim, Medius Klinik Kirchheim, University of Tübingen, Kirchheim-Teck, Germany

**Keywords:** proteinase 3-, myeloperoxidase-ANCA, methods, new recommendations, clinical significance

## Abstract

Anti-neutrophil cytoplasmic antibodies (ANCA) play a central role in the diagnosis and pathogenesis of patients with ANCA-associated vasculitis. ANCA-associated vasculitis is a rare disease characterized by necrotizing inflammation of small/medium-sized blood vessels with and without granuloma in different organs. The main syndromes are granulomatosis with polyangiitis, microscopic polyangiitis, and eosinophilic GPA. ANCA in these diseases are almost always directed against proteinase 3 and myeloperoxidase. Most laboratories worldwide use as standard the indirect immunofluorescence technique to screen for ANCA and then confirm positive IFT results with antigen specific immunoassyas for PR3- and MPO-ANCA. New guidelines for ANCA testing have been developed based on a recent European multicentre study, and according to the revised 2017 international consensus recommendations, testing for ANCA in small vessel vasculitis can be done by PR3- and MPO-ANCA immunoassays, without the categorical need for IIF. The new testing strategy for ANCA in vasculitis directly identifies the ANCA target antigen and has a particular value for the AAV sub-classification. Recent studies have shown that AAV can be classified based on ANCA serotype. ANCA presence and the antigen specificity also may have important value as a prognostic factor and may serve as a guide for immunosuppressive therapy. The clinical utility of ANCA depends on the type of assay performed and the appropiate ordering of testing the right clinical setting. Accurate identification of all patients with AAV and the avoidance of misdiagnosis can be achieved using a “gating policy” based on clinical information given to the laboratory at the time of request.

## INTRODUCTION

The detection of anti-neutrophil cytoplasmic antibody (ANCA) as a diagnostic tool and marker of disease in Wegener’s granulomatosis was described in 1985 by Van der Woude and coworkers.^1^ The spectrum of diseases associated with ANCA has since increased.^2^ Two ANCA are highly associated markers for ANCA-associated vasculitis (AAV), the latter of which includes granulomatosis with polyangiitis (GPA [formerly known as Wegener’s]), microscopic polyangiitis (MPA), eosinophil granulomatosis with polyangiitis (EGPA), and primary pauci-immune crescentic glomerulonephritis, namely C-ANCA synonymous with cytoplasmic fluorescence and specificity for proteinase 3 (PR3-ANCA), and P-ANCA with perinuclear fluorescence and specificity for myeloperoxidase. However, ANCA has limited specificity as it can be demonstrated in patients with inflammatory bowel disease (IBD), autoimmune liver disease, connective tissue diseases, infections and drug-induced vasculitis, often with multiple antigen specificities and unclear clinical significance.^2,3^

**Figure 1:**
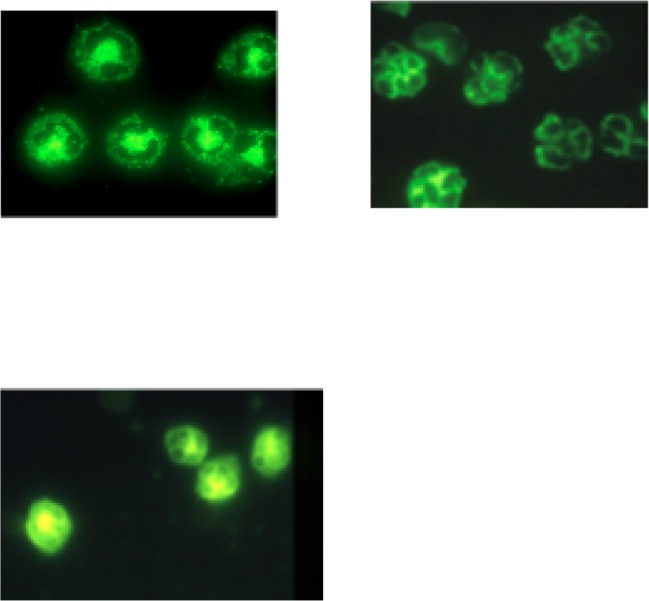
ANCA main fluorescence patterns: C-ANCA (cytoplasmic) ANCA, P-ANCA (perinuclear) and A-ANCA (atypical) on ethanol-fixed neutrophils

Detection of ANCA in vasculitis is based on primary screening by immunofluorescence test (IFT) on ethanol-fixed neutrophils, and positive indirect immunofluorescence (IIF) test should always be followed by specific PR3- and MPO-ANCA immunoassays (**Figure 1**). Ideally all three tests should be used in each sample.^4,5^ Since the establishment of this consensus, many new antigen specific immunoassays have become available, and this has challenged the position of IFT in the testing algorithm for ANCA in vasculitis.^6,7^ Furthermore, there is a need for screening algorithm including clinical gating policies to guide the flow of analysis in diagnostic laboratories. In the right clinical context, a positive test for PR3- or MPO-ANCA has high sensitivity and specificity for a diagnosis of AAV.

This review considers current data on ANCA testing, presents the new international consensus on ANCA testing, and discusses the usefulness of PR3- and MPO-ANCA in the diagnosing and managing of patients with small-vessel vasculitis.

## PARADIGM SHIFT IN ANCA DIAGNOSTIC: NEW INTERNATIONAL CONSENSUS RECOMMENDATIONS

An international consensus statement for ANCA testing was issued in 1999 and states that “*ANCA is best demonstrated by using a combination of indirect immunofluorescence (IIF) and enzyme-linked immunosorbent assays (ELISAs) that detect ANCA specific for proteinase 3 (PR3) or myeloperoxidase (MPO). For ANCA testing in “new” patients, IIF must be performed on all serum samples*. *Serum samples containing ANCA […] should be tested in ELISAs for PR3-ANCA and MPO-ANCA*”.^4^ Although this consensus is still widely applied, the position of IIF is being questioned.

Over the last 15 years, the performance of ELISA has improved and novel, sensitive and automated technologies, such as fluoro-enzyme immunoassay, chemiluminescence assay and multiplexed flow immunoassay, have been introduced. Besides, there have been advances in assay setup (antigen presentation) with development of second (capture-based) and third (anchor-based) generation assays. Largely, currently available assays for PR3-ANCA and MPO-ANCA are highly sensitive and specific for GPA and MPA.^7^

The availability of reliable antigen-specific immuno-as-says has raised doubts about the two-stage diagnostic strategy currently recommended for ANCA detection.^6,7^ In a recent European Vasculitis Study (EUVAS) multicenter study, it has been confirmed that the diagnostic performance of antigen-specific immuno-assays equaled or even exceeded the diagnostic performance of IIF.^8,9^ When compared to the study of Hagen and colleagues which was 20 years ago and was a major basis for the consensus statement, there have been marked improvements (mainly in specificity) of the antigen-specific immunoassays.^10^

Given these improvements, the revised international consensus demonstrated that high quality antigen-specific immunoassays can be used for the diagnosis of ANCA-associated vasculitis without the need for IIF.^11^ Furthermore, it was shown that appropriately designed reference ranges for antibody levels improve interpretation of antigen-specific immuno-assays.^12^ When new assays are introduced (and for assays not included in the above-mentioned international study, diagnostic performance characteristics need to be checked based on diagnostic samples of GPA/MPA patients and relevant disease controls.^8,9^ One needs, however, to realize that single immuno-assays never have a sensitivity and specificity of 100%. In case of a high clinical suspicion and a negative test result, a second immuno-assay or IIF can be used to increase the sensitivity. Performing a second assay or IIF can also (marginally) increase the specificity in case of (low) positive test results. Alternatively, if IIF is used as a screening assay in locally determined best testing algorithms, then the laboratory needs to ensure that the IIF operates at a high sensitivity as there is a large variability in performance of IIF between laboratories.

Whereas PR3- and MPO-ANCA have been shown to be highly specific for AAV, their diagnostic value of non-vasculitic conditions is very limited. An overestimation of the diagnostic relevance of a positive ANCA test may erroneously misdirect clinicians and delay adequate treatment. The diagnostic accuracy of ANCA testing in vasculitis should be improved by an increased pretest probability.^11^

Accurate identification of all patients with AAV and the avoidance of misdiagnosis can be achieved by use a “gating policy” based on clinical information given to the laboratory at the time of request. This policy limits requests for ANCA testing exclusively to clinical scenarios that may suggest a diagnosis of necrotizing vasculitis.^11^ Recently, Arnold and colleagues investigated the impact of a “gating policy” at a single regional center in the year prior to and following the consensus guidelines, and demonstrated that adherence to a “gating policy” for ANCA testing coupled with close liaison between clinician and laboratory does not result in either a missed or delayed diagnosis of AAV.^13^

In summary, the new recommendation is that PR3- and MPO-ANCA immunoassays can be used for accurate diagnosis of ANCA-associated vasculitis, without the need for IIF.^11^ The current consensus recommendation applies to ANCA testing for the diagnosis of vasculitis, but does not apply to ANCA testing for the diagnosis of inflammatory bowel disease, autoimmune liver disease or drug-induced autoimmunity.

## PR3- AND MPO-ANCA ARE USEFUL TOOL FOR DISEASE CLASSIFICATION IN SMALL VESSEL VASCULITIS

Existing classification systems of small vessel vasculitis have relied on combinations of different clinical, radio-graphic and histological findings, but have not included ANCA specificity. Accumulating evidence suggests that ANCA specificity could be better than clinical diagnosis for defining groups of patients, as PR3- and MPO-ANCA are associated with different genetic backgrounds, epidemiology, clinical features, histological findings, and pathogenesis. The new classification approach of AAV is to use ANCA serotypes to classify disease and provide immediate diagnosis based on the presence of PR3- and/or MPO-ANCA. It was demonstrated that serotyping distinguishes distinct classes of ANCA disease: PR3-ANCA associated Vasculitis (PR3-AAV), MPO-ANCA associated Vasculitis (MPO-AAV) and ANCA-negative Vasculitis.^14^

The genetic data from genome wide studies (GWAS) point to genetic differences between PR3- and MPO-ANCA patients. These studies have shown that the autoantigen specificities PR3 and MPO correlate better with different HLA risk genes (PR3-ANCA with HLA-DP, MPO-ANCA with HLA-DQ) than with the clinical and pathological phenotypes of GPA and MPA.^15^ Moreover, the association with the genes encoding PR3 (PTN3) and its inhibitor α-1-antitrypsin (SERPINA1) with PR3-ANCA disease and/or GPA additionally supports a central pathogenetic role of these autoantigens and their neutralizing counterparts.^15^

Clinical features differ both in the type and in frequency between PR3-AAV and MPO-AAV. PR3-AAV is associated with granulomatous inflammation, respiratory tract involvement, more extensive extra-renal organ manifestation and a higher relapse rate.^16,17^ In contrast, MPOAAV is more frequent in kidney-limited disease, displays more severe renal scarring and a worse renal prognosis. Furthermore, patients with MPO-ANCAs are more likely to have renal pathology classified as mixed or sclerotic, and to have a strong association with lung fibrosis compared to patients with PR3-ANCA.^18,19^

Among patients receiving a clinical diagnosis of GPA, those positive for MPO-ANCAs more frequently have limited disease without severe organ involvement, a higher prevalence of subglottic stenosis, a less frequent need for cyclophosphamide or rituximab therapy and fewer relapses than patients who are positive for PR3ANCA.^20,21^ These data indicate that ANCA specificity is a major determinant of the clinical presentation in AAV.

In summary, the classification of patients by ANCA specificity (PR3-ANCAs versus MPO-ANCAs) provides practical diagnostic criteria better aligned to patient phenotype, outcomes and treatment responses than does their classification by clinical diagnosis (GPA vs. MPA and EGPA).^14^

## CONCLUSION

ANCA serology and the associated clinical manifestation of AAV continue to attract the attention of clinicians and investigators alike. In addition to be providing a useful diagnostic tool for small vessel vasculitis, ANCA testing may be useful in predicting relapses and in guiding therapy. The revised consensus recommendations on ANCA testing state that high quality antigen-specific immunoassays are the preferred screening methodology for the diagnosis of ANCA-associated vasculitis. IIF is no longer deemed suitable as the first screening test, and it adds little to antigen-specific assays in the diagnosis of ANCA-associated vasculitis when the pre-test probability of the disease is high.

Notwithstanding, any testing strategy applicable to vasculitis should be able to identify relevant ANCA target antigens (PR3 and MPO), since these correlate best with the clinicopathological aspects, disease activity, propensity for relapse, and response to therapy.
